# Advances in the Study of Chronic Active Epstein-Barr Virus Infection: Clinical Features Under the 2016 WHO Classification and Mechanisms of Development

**DOI:** 10.3389/fped.2019.00014

**Published:** 2019-02-05

**Authors:** Ayako Arai

**Affiliations:** Department of Laboratory Molecular Genetics of Hematology, Graduate School of Medical and Dental Sciences, Tokyo Medical and Dental University (TMDU), Tokyo, Japan

**Keywords:** epstein-barr virus, chronic active EBV infection, NF-κB, STAT3, T-or NK-lymphoproliferative disease, inflammation, hematopoietic stem cell transplantation, lymphoma

## Abstract

Chronic active Epstein-Barr virus infection (CAEBV) is one of the Epstein-Barr virus (EBV)-positive T- or NK-lymphoproliferative diseases. It is considered rare and geographically limited to Japan and East Asia. However, CAEBV is drawing international attention, and the number of case reported worldwide is increasing, after its classification in the EBV-positive T- or NK-cell neoplasms, in the 2016 WHO classification. In this article, I review current advances in the study of CAEBV under the new definition and show future directions. In CAEBV, EBV-infected T or NK cells clonally proliferate and infiltrate multiple organs, leading to their failure. These characteristics define CAEBV as a lymphoid neoplasm. However, the main symptom of CAEBV is inflammation. Recently, the mechanisms underlying the development of CAEBV have gradually become clearer. EBV infection of T or NK cells can occur during the acute phase of primary infection with a high EBV load in the peripheral blood. In addition, it was reported that cytotoxic T cells decreased in numbers or showed dysfunction in CAEBV. These findings suggest that undetermined immunosuppressive disorders may underlie persistent infection of T or NK cells. Furthermore, EBV itself contributes to the survival of host cells. *In vitro* EBV infection of T cells induced intercellular survival-promoting pathways. Constitutive activation of NF-kB and STAT3 was observed in EBV-positive T or NK cells in CAEBV, promoting not only cell survival but also CAEBV development. During the disease course, CAEBV can lead to two lethal conditions: hemophagocytic lymphohistiocytosis and chemotherapy-resistant lymphoma. It is necessary to start treatment before these conditions develop. At present, the only effective treatment strategy for eradicating EBV-infected T or NK cells is allogeneic stem cell transplantation (allo-HSCT). However, patients with an active disease, in which the condition is accompanied by fever, liver dysfunction, progressive skin lesions, vasculitis, or uveitis, had worse outcomes after allo-HSCT, than patients with an inactive disease had. Unfortunately, current chemotherapies are insufficient to improve the activity of CAEBV. Based on the molecular mechanisms for the development of the disease, the NF-kB, or JAK/STAT mediating pathways are attractive candidate targets for new treatments.

## Introduction

Epstein-Barr virus (EBV) is a ubiquitous double-stranded DNA virus, categorized under the human herpes virus family. The virus was discovered in 1964 in cells affected by endemic Burkitt lymphoma (1). Once EBV infects human beings, it cannot be eradicated and latently infects B cells throughout the lifespan. EBV immortalizes infected B cells and can be a cause of B-cell neoplasms under immunocompromised conditions, which can promote the proliferation of EBV-infected B cells. The vast majority of immunodeficiency-associated lymphoproliferative disorders are classified into these categories.

B cells are not the only targets of EBV. Epithelial cells may also test positive for the EBV genome, leading to the development of nasopharyngeal cell carcinoma. Furthermore, the EBV genome is also positive in T- or NK-lymphoid neoplasms. Chronic active EBV infection is one of the EBV-positive T- or NK-lymphoproliferative diseases (EBV-T/NK-LPDs). It was originally reported in Western countries but has been primarily reported and studied in Japan and neighboring countries. In 2016, CAEBV was classified under EBV-positive T- or NK-cell neoplasms in the revised WHO classification of tumors of hematopoietic and lymphoid tissues (2). Since then, CAEBV has been drawing international attention, and the number of case reports on the topic is increasing worldwide. Although CAEBV can be lethal, some patients have recently achieved long-term survival by allogeneic hematopoietic stem cell transplantation (allo-HSCT). Furthermore, the mechanisms by which EBV infects T or NK cells in the small number of patients who develop CAEBV are currently being clarified. In this review, I describe the current status of CAEBV based on its new definition, focusing on the mechanisms underlying its development, the diagnostic and therapeutic procedures for the disease and future directions.

## History of CAEBV and Its Related Disorders

To the best of my knowledge, the first report of suspected CAEBV was in the U.S. in 1948 (3), documenting 53 cases with fever and splenomegaly “from 3 months to longer than 4 years after the initial attack” as chronic infectious mononucleosis (IM). Three of the patients developed “lymphoblastoma.” Subsequently, other researchers reported similar cases (4). They considered the disease to be sustained IM and named it CAEBV. However, this disease was not equivalent to sustained IM. In 1988, Jones and colleagues reported that EBV-infected and clonally proliferating T cells were detected in CAEBV (5). Similar reports followed, mainly from Japan and East Asia. These reports indicated that EBV-positive NK cells were also detected in CAEBV. After the 1980s, it was confirmed that CAEBV was a progressive disease and that EBV-infected cells infiltrate multiple organs, leading to their dysfunction. Furthermore, conditions with characteristic skin lesions, such as severe mosquito bite allergy (sMBA) or hydroa vacciniforme (HV), have EBV-infected T or NK cells and show disease courses similar to that of CAEBV. In 2005, Okano et.al suggested the first diagnostic guidelines for CAEBV: persistent and recurrent IM-like symptoms; an unusual pattern of anti-EBV antibodies, with raised levels of anti-VCA and anti-EA; detection of increased EBV genomes in affected tissues, including the peripheral blood (PB); and chronic illness that cannot be explained by other known disease processes at diagnosis (6). It also mentioned that hemophagocytic lymphohistiocytosis (HLH) or LPD/lymphoma originated from the T- or NK-cell lineage, often developed during the disease course. It should be noted that the guidelines clearly defined CAEBV as a disease distinct from known immunosuppressive conditions. Three years later, Ohshima and colleagues reported that CAEBV patients showed clonal evolution of the infected cells and eventually developed T- or NK-cell lymphoma or leukemia from the viewpoint of pathologist (7). They suggested multistage lymphomagenesis of EBV-T/NK-LPDs. Subsequently, the WHO classification of tumors of hematopoietic and lymphoid tissues, which was revised in 2008, first described CAEBV as a systemic EBV-T-LPD of childhood (8). This was a major event that identified CAEBV as a neoplastic disorder. However, the classification had some issues. First, CAEBV with an EBV-infected NK-cell type was not described. Second, the term childhood reminds us that the disease is a pediatric disorder. Kimura and colleagues performed a prospective assay of 108 patients with EBV-T/NK-LPDs, which was accompanied by sustained inflammation with the EBV infection of T or NK cells (9). They categorized these disorders into 4 subtypes: CAEBV, sMBA, HV-like lymphoproliferative disorder (HV-LPD), and EBV-associated hemophagocytic lymphohistiocytosis (EBV-HLH). In the report, CAEBV was described as a disease harboring systemic inflammation, and sMBA and HV-LPD were defined as diseases with lesions limited to the skin. According to these studies, the new WHO classification revised in 2016 defined CAEBV as an EBV-T/NK-LPD (2). In Japan, the research group, Measures against Intractable Diseases by the Ministry of Health, Labor and Welfare of Japan, suggested that the diagnostic criteria for CAEBV with EBV infection of T- or NK cells be added to Okano's guidelines (Table 1). The criteria matched the new WHO classification.

**Table 1:** Diagnostic criteria of Chronic active Epstein-Barr virus infection.

(1) Sustained or recurrent IM-like symptoms persist for more than 3 months
(2) Elevated EBV genome load in the peripheral blood (PB) or the tissue lesion
(3) EBV infection of T or NK cells in the affected tissues or the PB
(4) Exclusion of other possible diagnoses: primary infection of EBV (infectious mononucleosis), autoimmune diseases, congenital immunodeficiencies, HIV, and other immunodeficiencies requiring immunosuppressive therapies or underlying diseases with potential immunosuppression
Patients who fulfilled criteria (1–4) were diagnosed with CAEBV.

*(1) IM-like symptoms generally include fever, swelling of lymph nodes, and hepatosplenomegaly; additional complications include hematological, gastroenterological, neurological, pulmonary, ocular, dermal, and/or cardiovascular disorders (including aneurysm and valvular disease), which have mostly been reported in patients with IM. EBV-HLH accompanied by primary infection of EBV and HV, whose symptoms are limited to those in the skin, should be excluded. Even if EBV-HLH or EBV-positive T- or NK-cell lymphoma/leukemia develops during the disease course, the original diagnosis of CAEBV does not change*.

*(2) A standard for elevated EBV DNA load by quantitative PCR in the PB is more than 10*^2.5^ *copies/*μ*g DNA*.

*(3) For detection of EBV-infected cells, it is recommended to perform a combination analysis of detecting the phenotypes of the infected cells (immune fluorescent staining, immune histological staining, magnetic bead sorting) and detecting EBV (EBNA staining, EBV-encoded small RNA in situ hybridization, PCR for EBV DNA)*.

*(4) Patients who were diagnosed with congenital immune deficiencies, autoimmune diseases, collagen diseases; patients who were pathologically diagnosed with malignant lymphomas [Hodgkin lymphoma; extranodal NK/T-cell lymphoma, nasal type (ENKL); angioimmunoblastic T-cell lymphoma; peripheral T-cell lymphoma (PTCL); aggressive NK-cell leukemia (ANKL)]; and patients who were diagnosed with an iatrogenic immunosuppressive condition, either concurrently or prior to CAEBV diagnosis, were also excluded from CAEBV*.

Cohen and colleagues, an American research group, reported 19 patients with CAEBV in the U.S (10). The types and frequencies of EBV-infected cells were as follows: T or NK cells, 4, B cells 11, T cells 3, and NK cells 1. Thus, the majority had B-cell type. Interestingly, most patients with B-cell type also had hypogammaglobulinemia prior to B-cell suppressing therapy, such as rituximab or cytotoxic reagents, whereas T- or NK-cell type patients did not. Thus, B-cell type CAEBV may be a different disorder from T- or NK-cell type CAEBV.

## Epidemiology of CAEBV

**Figure 1:**
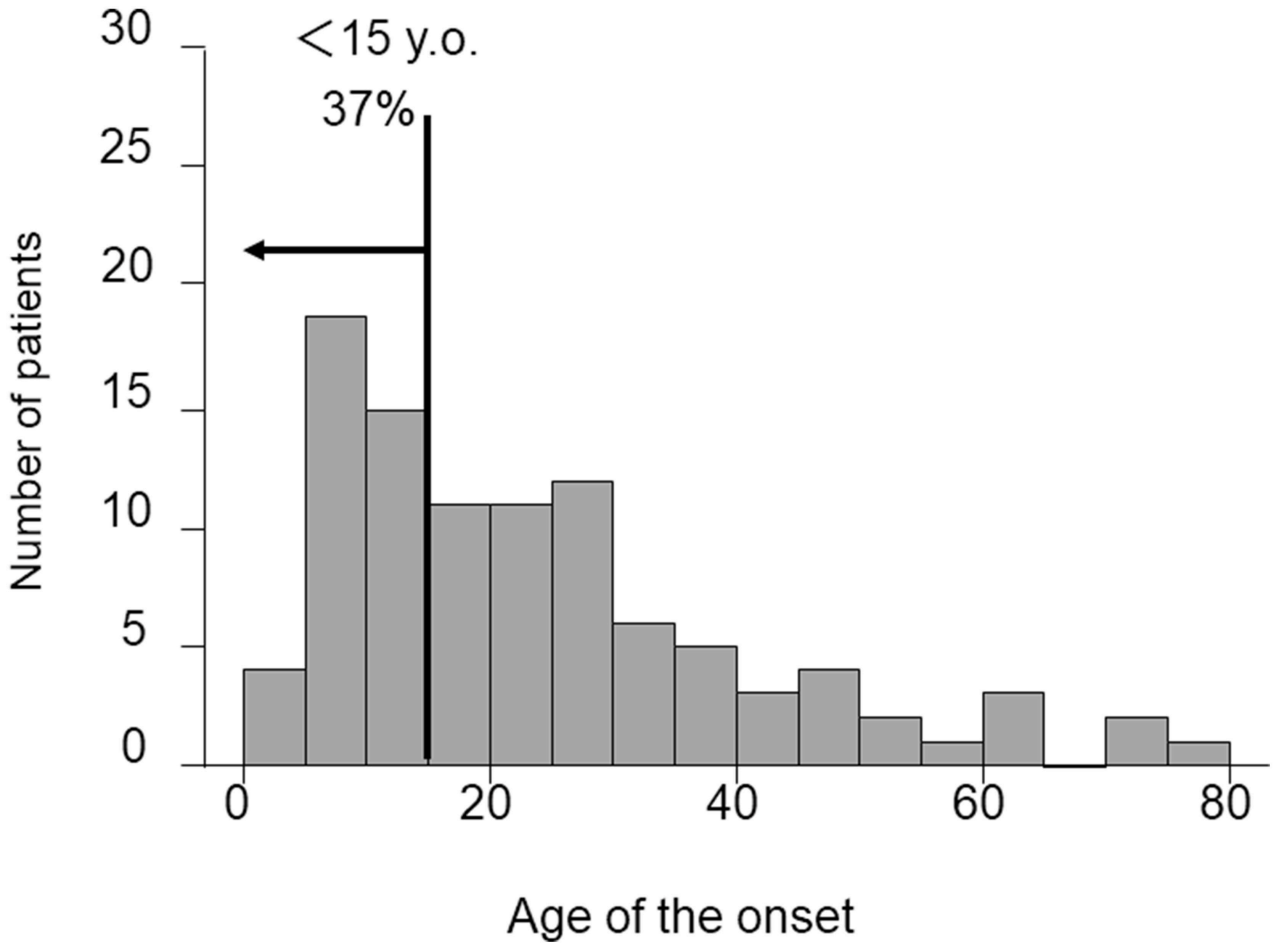
Distribution of age at diagnosis of CAEBV patients. Patient data were collected by means of a nationwide survey of the Japanese study group of the Japan Agency for Medical Research and Development, AMED. Patients had been newly diagnosed with CAEBV between January 2003 and March 2016.

The rate of the onset of CAEBV in Japan was 23.8/year, according to the annual report of the research group of Measures against Intractable Diseases by the Ministry of Health, Labor and Welfare of Japan. CAEBV had been considered a childhood disease, as mentioned previously. However, as shown in Figure 1, the Japanese nationwide CAEBV survey showed that more than half of the patients were adults. Several reports indicated that the prognosis of adult-onset patients was poorer than that of childhood-onset patients, suggesting that adults and children might have different disorders (9, 11, 12). In addition, most cases to date have been reported in Japan and East Asia. Some investigators have suggested that there are genetic issues in the development of CAEBV (13). However, there has been little evidence to support this hypothesis. Further studies with a large number of patients are necessary to determine the detailed epidemiology of CAEBV.

## Clinical Features of CAEBV

CAEBV has two characteristics: systemic inflammation and neoplastic disease. In CAEBV, EBV-infected T or NK cells clonally proliferate and infiltrate systemic organs, leading to their failure. These characteristics define CAEBV as a lymphoid neoplasm. However, CAEBV rarely has solid tumors. In fact, the main clinical finding of CAEBV is inflammation. According to Kimura's report, major clinical findings of CAEBV were fever, liver dysfunction, thrombocytopenia, anemia, and their incidences were 91, 77, 59, 44, and 43%, respectively (9). Furthermore, CAEBV causes vasculitis due to the direct invasion of the infected cells, as well as an immune reaction caused by activation of the cells. Every organ can be a target. Vasculitis can lead to the development of vascular aneurysms, ischemic organ damage and uveitis. As a result, patients with CAEBV can visit any hospital department. Every clinician should be aware of CAEBV and consider it a differential diagnosis in cases of sustained inflammation of unknown origin.

Two skin conditions are well-known CAEBV-related disorders. sMBA is characterized by local skin inflammation followed by high fever, lymphadenopathy and liver dysfunction following the bites of *Aedes (Stegomyia) albopictus*—also known as the Asian tiger mosquito. The puncture sites ulcerate, and although they can be cured within a month, they often leave scars. sMBA occurs due to the hyperreactive response of the patients' lymphocytes to the mosquito's saliva (14). In 1997, Ishihara et al. detected a monoclonal proliferation of EBV-positive T and NK cells in the PB of sMBA patients (15). The following reports indicated that sMBA could lead to the development of fatal disorders such as T- or NK-cell lymphoma or HLH. Therefore, sMBA is now classified as one of the EBV-T/NK-LPDs (2). HV, which is characterized by light-induced vesicles, can be accompanied by systemic inflammation with detection of EBV-infected clonally proliferating T or NK cells (16), and was defined as an HV-LPD in WHO 2016 (2). The diagnoses of sMBA and HV-LPD are made for conditions in which the lesions are limited to the skin. Some CAEBV patients have hyper sensitivity for mosquito bites or HV-like eruptions. The difference of pathogenesis between these skin-limited diseases and CAEBV has not been clarified due to the rarity of these diseases. The analysis of a large number of patients under the unified diagnostic criteria is critical to better understand these diseases.

CAEBV is a progressive disease with two characteristics, specifically, systemic inflammation and the development of neoplasms during the disease course, leading to two lethal conditions, namely, HLH and chemotherapy-resistant lymphoma, respectively. The duration from disease onset to the development of these conditions ranges from several months to several decades. Establishing how to predict and prevent the development of these conditions is an urgent issue.

## Diagnostic Procedures for CAEBV

**Figure 2:**
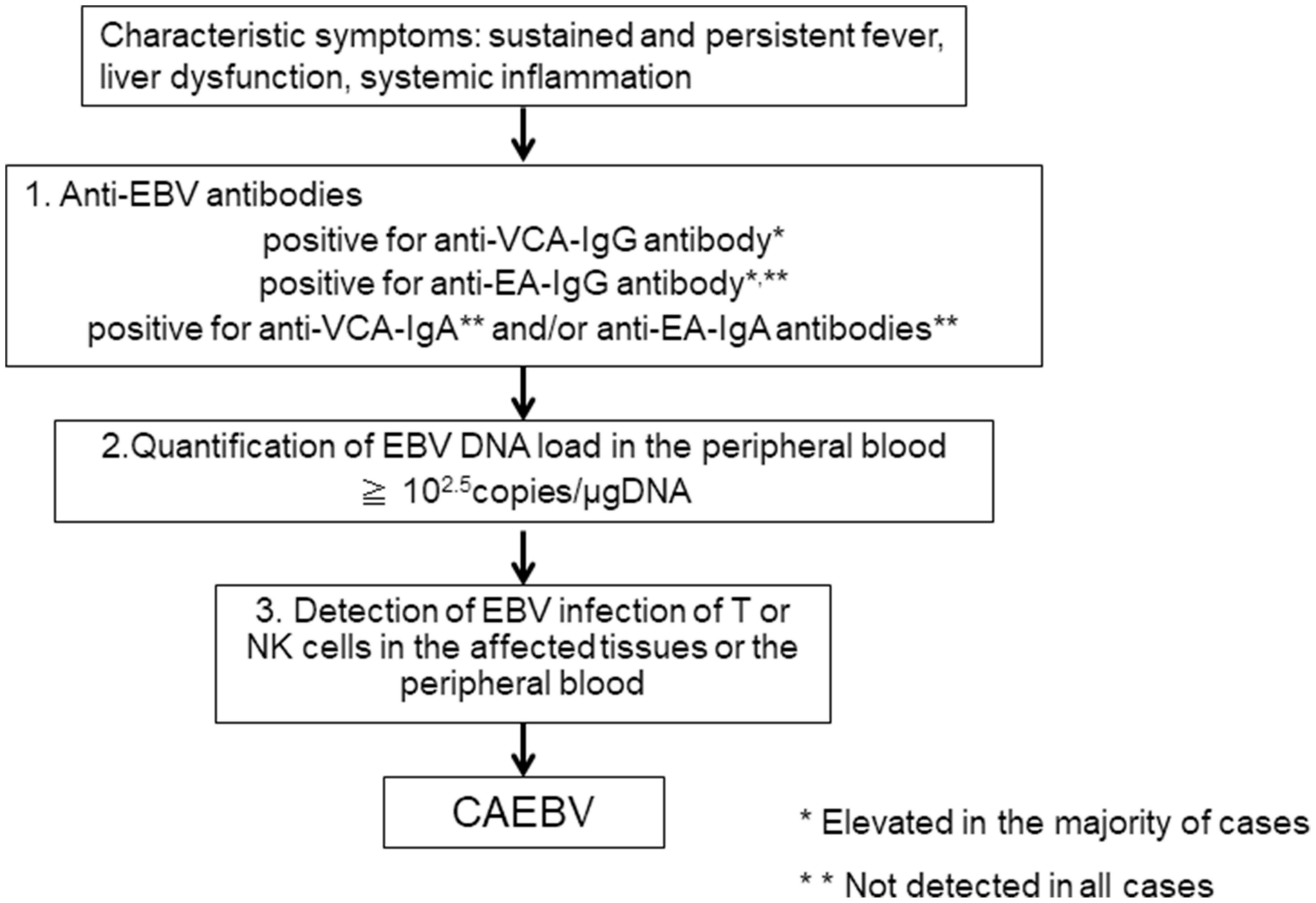
A flowchart of the diagnosis of chronic active EBV infection.

When clinical doctors see patients suffering from sustained inflammation, CAEBV as a differential diagnosis is rarely considered, because they are either unaware of the disease or because of the rarity of the disease. According to our report, the mean length of time from onset to diagnosis in CAEBV patients was 20 months (11). The first step to correctly diagnosing CAEBV is to suspect the disease. CAEBV should be frequently considered in cases of sustained inflammation of unknown origin. Table 1 presents the diagnostic criteria of CAEBV suggested by the Japanese study group in 2015, based on previous reports. Figure 2 shows a flow chart of the diagnosis of CAEBV. It is not difficult to make a diagnosis of CAEBV under the criteria and the procedure. However, some issues need to be resolved.

### Anti-EBV Antibodies

If CAEBV is suspected, anti-EBV antibodies should first be examined. An anti-VCA-IgG antibody is necessary to confirm EBV infection. Many CAEBV cases show high anti-VCA-IgG antibody levels of more than × 640 (6). According to the previous guidelines for the diagnosis, the majority of cases show high levels of anti-EA-IgG antibody and positive for anti-VCA-IgA and anti-EA-IgA antibodies that are originally positive in the acute phase of primary EBV infection (6). Therefore, it is important to rule out primary infection of EBV, IM. The clinical and laboratory findings of IM and CAEBV are quite similar and sometimes difficult to distinguish. Importantly, most IM cases resolve spontaneously, whereas CAEBV needs intensive treatment, including allo-HSCT, to be cured. An anti-EBNA antibody is not useful because some cases of CAEBV are negative for anti-EBNA antibody. An anti-VCA IgM antibody is more useful to exclude primary infection of EBV, but it is not infallible. It is important to check a patient's accurate clinical history to rule out primary EBV infection.

### EBV-DNA Load in the Peripheral Blood

If a patient with sustained inflammation of an unknown cause shows elevated levels of anti-VCA-IgG, anti-EA-IgG antibodies, and is positive for anti-VCA-IgA or anti-EA-IgA antibodies, and if IM can be ruled out, the next step to making a diagnosis of CAEBV is the quantification of the EBV DNA load in the PB by PCR. In CAEBV, EBV-infected T or NK cells can be detected in the PB and are characteristic of the disease. Therefore, EBV DNA is detected in the fraction containing mononuclear cells in the blood (17). A cut-off value for the EBV DNA load in the PB for CAEBV is 10^2.5^ copies/μgDNA (18), and the EBV DNA load can become undetectable after successful allo-HSCT (19). On the other hand, EBV DNA is usually detected in the serum of EBV-positive lymphomas, including the following: extranodal NK/T-cell lymphoma, nasal type (ENKL), EBV-positive Hodgkin lymphoma; and nasopharyngeal cell carcinoma (20–22). In 2018, the quantification of the EBV DNA load in the PB by RT-PCR was approved as an examination of CAEBV and is covered by health insurance in Japan.

### Detection of EBV-Infection of T or NK Cells

If EBV DNA is detected in PB-containing mononuclear cells, the next step is to detect EBV infection of T or NK cells in the affected tissues or the PB. If pathological specimens of EBV-infected cell infiltrating organs are available, histological examination by immune staining and *in situ* hybridization of Epstein-Barr virus-encoded mRNA (EBER) is performed to detect the phenotypes. However, CAEBV rarely develops solid tumors. As mentioned above, EBV-infected cells can be detected in the PB of CAEBV. Therefore, the phenotypes of EBV-infected cells were determined using unfixed PB in most patients (9, 11, 23). This procedure is costly and requires skilled examiners. In addition, institutes that are capable of performing the examination are limited. This issue is serious and makes the diagnosis of CAEBV difficult. It is indispensable to establish more convenient procedures to determine phenotypes of EBV-infected cells.

## The Suggested Mechanisms of the Development of CAEBV

EBV is a common virus; almost all adults have been infected with the virus worldwide. Why does EBV infect T or NK cells, which leads to the development of CAEBV in specific patients? Recently, the mechanisms have gradually become clearer.

There has been a geographical concentration of the reports of CAEBV in Japan and East Asia, indicating that CAEBV is an Asian endemic disorder and that patients may have a common genetic background. However, this hypothesis is controversial. In Western countries, CAEBV patients certainly exist, even in the Caucasian population. Currently, members of the Japanese study group are investigating genetic factors contributing to the development of CAEBV using next-generation sequencing.

How does EBV infect T or NK cells? EBV infects its target B cells by associating with CD21 on the cell surface as a receptor. It has been reported that weak expression of CD21 can be detected on T cells (24). In addition, an *in vitro* examination reported that activated NK cells that were conjugated to CD21-positive EBV-infected B cells transiently acquired weak CD21 expression by the synaptic transfer of a few receptor molecules onto their surface (25). A similar mechanism also exists in T cells (26). Furthermore, another *in vitro* infection assay using a high EBV load showed that EBV infection of T or NK cells could be established (27, 28). Additionally, *in vivo* EBV infection of T or NK cells can be detected in the rapid phase of IM patients. These findings indicated that under a high viral load, EBV can infect T or NK cells (29). Although it is unknown whether the infection is transient or the appearance of the infected cells is transient, EBV-positive T cells disappeared 1 year after onset in IM (30). Why can EBV infection of T or NK cells be sustained in CAEBV? Two mechanisms can be suggested: suppressed immune reaction to the infected cells or characteristics of the virus. It was reported that cytotoxic T cells (CTL) decreased in numbers or showed dysfunction in CAEBV (31, 32). In addition, some congenital immunosuppressive disorders, such as the case of autoimmune lymphoproliferative disorder (ALPS) with FAS gene mutation (33) or the case of perforin mutation (34), can be complicated by CAEBV-like conditions. CAEBV is not accompanied by known primary immunodeficiency disorders (35), however undetermined immunosuppressive disorders may co-occur. Virus-related factors may also play a role. Although characteristic viral strains of CAEBV have not yet been determined, Japanese groups are currently working to clarify these issues through genome-wide analyses. The results are highly anticipated.

EBV infects B cells and immortalizes them. The next question is how EBV-infected T or NK cells become neoplastic cells. Several studies have reported that survival-promoting molecules or pathways are activated by EBV infection. Imadome et al. found that EBV-infected T or NK cells obtained from CAEBV patients expressed CD40 (36). They performed *in vitro* EBV infection of T cells and observed inducible CD40 expression on the surface (27). CD40 was originally expressed on activated B cells. Because the ligand of CD40, CD40L, is originally expressed on the surface of activated T cells, it was hypothesized that inducible CD40 expression was associated with CD40L on the T-cell surface and activated intracellular signaling molecules such as NF-kB. They also confirmed that CD40 on EBV-infected T cells activated CD40L-mediating signaling in an autocrine or paracrine manner and suppressing their apoptosis (27). Another costimulatory molecule, CD137, may contribute to promoting the survival of EBV-infected T cells. Yoshimori et al. reported that CD137 was also expressed in EBV-infected T or NK cells in CAEBV, and its expression could be induced by *in vitro* EBV infection on T cells (23). Stimulation of CD137 by CD137 ligand suppressed etoposide-induced cell apoptosis. Furthermore, Takada et al. found that NF-κB, a transcription factor mediating cell survival signals, was constitutively activated in EBV-infected T or NK cells in CAEBV (37). They also reported that *in vitro* EBV infection of T cells induced constitutive activation of NF-κB and suppressed serum depletion and etoposide-induced apoptosis of the infected cells. NF-κB exists downstream of CD40 and CD137. These findings suggest that EBV infection directly induces cell survival of T or NK cells via survival-promoting pathways such as NF-κB.

EBV may contribute not only to promoting cell survival but also to inducing gene mutations in EBV-infected cells. Nakamura et al. observed activation-induced cytidine deaminase (AID) in the peripheral blood mononuclear cells of EBV-T/NK-LPD patients (38). AID is essential for the somatic hypermutation and class switch recombination of immunoglobulin genes (39). Deregulated AID expression acts as a genomic mutator, leading to the development of B-cell lymphoma (40). In addition, EBV infection induces AID expression in B cells (41). These findings suggest that AID plays a role in EBV-induced lymphomagenesis in B cells. Further studies are expected to determine whether AID has the same roles in CAEBV development.

Recently, interesting findings have been reported by some investigators using next generation sequencing. Okuno et al. performed whole-exome sequencing (WES) on T-, B-, and NK-cell subsets from CAEBV patients. They reported that the most frequently mutated gene was *DDX3X*, an RNA helicase gene detected in 16% (14/83) (42). They also reported that patients carrying *DDX3X* mutation at diagnosis demonstrated significantly shorter overall survival (OS) in comparison with patients without the mutation. Interestingly, Jiang et al. preformed WES for tumor cells from ENKL and reported that *DDX3X* was frequently mutated in them (20%, 21/105) (43). They also determined that the mutant exhibited growth promoting effects on NK cells in comparison with the wild-type protein. *DDX3X* mutation was also detected in Burkitt lymphoma (44). Many reports have focused on *DDX3X* and their association with cancers (45), and the mutation has a possibility of a common driver mutation of EBV-positive neoplasms. Other than *DDX3X*, however, various mutations were detected in CAEBV by Okuno et.al: *KMT2D* (4.8%), *BCOR*/*BCORL1* (3.6%), *KDM6A* (3.6%), and *TET2* (2.4%) (42). Furthermore, they reported the detection rate of at least one somatic mutation in CAEBV by WES was 52% as a whole. These findings indicate a diverse background of CAEBV.

CAEBV has common characteristics of inflammatory disorders. In patients with CAEBV, the serum levels of inflammatory cytokines, namely, IFN-γ, TNF-α, and IL-6, are higher than those in healthy people (46, 47). Their elevated serum levels are associated with the status of the disease. In addition, Onozawa et al. reported that the mRNA of these cytokines was increased in EBV-infected T or NK cells obtained from CAEBV patients (46). The expression of these inflammatory cytokines can be induced by NF-κB (48). Constitutive activation of NF-κB in CAEBV may contribute to the production of cytokines.

STAT3 is a transactivation factor that mediates proliferation and anti-apoptotic signaling. It is activated in various cancer cells and contributes to their transformation (49). STAT3 also mediates intracellular signaling downstream of cytokines and regulates inflammation (50). We observed that STAT3 was constitutively activated in EBV-positive T or NK cells, not only in EBV-positive T or NK cell lines established from EBV-T/NK-lymphoid neoplasms, but also in neoplastic EBV-positive T or NK cells obtained from CAEBV patients (51). Since there was no mutation in the SH2 domain of the STAT3 gene, an essential site for activation of the molecules serving as hot spots of activating mutations in other T or NK cell tumors, upstream molecules might contribute to the activation. Several studies have suggested that STAT3 is activated downstream of LMP1 through the activation of NF-κB (52, 53). Interestingly, Onozawa et al. found that inhibition of a tyrosine kinase, JAK1/2, which phosphorylates STAT3 by its inhibitor ruxolitinib, inhibited STAT3 activation in EBV-positive T or NK cell lines. Furthermore, ruxolitinib suppressed proliferation and induced apoptosis of these cells (51). It was also determined that ruxolitinib suppressed the mRNA expression of IFN-γ, TNF-α, and IL-6 in EBV-T/NK cells. These results indicated that the JAK1/2 STAT3 pathway contributed to the development of both the inflammatory and neoplastic aspects of CAEBV.

**Figure 3:**
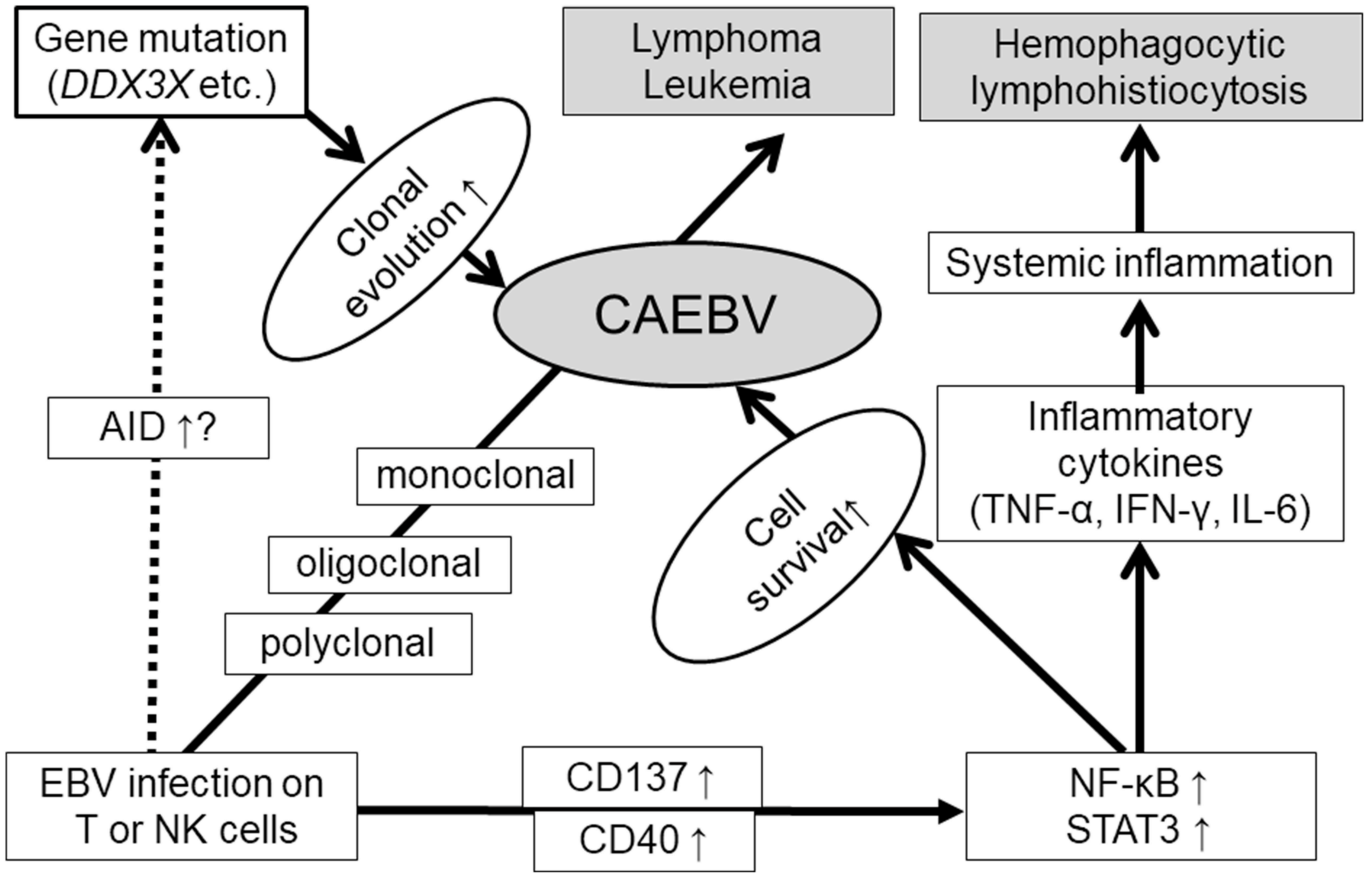
Suggested mechanisms of CAEBV development.

In summary, EBV infection of T or NK cells can occur during the acute phase of primary infection with a high EBV load. Furthermore, EBV itself contributes to the survival of host cells by inducing CD40 and CD137 expression and constitutive activation of NF-κB in infected cells. The upregulated AID expression, and accumulation of gene mutations of the infected cells during the course were reported. Gain of function mutation of *DDX3X* may be a responsible driver mutation. Once driver mutations occur, high malignant disorders, such as lymphoma or leukemia, can develop. Furthermore, constitutive activation of STAT3 promotes not only cell survival but also the production of inflammatory cytokines. These suggested mechanisms of CAEBV development are summarized in Figure 3.

## Current Treatment Strategy for CAEBV

The purpose of the treatment for CAEBV is to control two faces of the disease: a neoplasm and an inflammatory disease. Once HLH or lymphoma develops, CAEBV can be fatal. Therefore, it is recommended that treatment starts before these diseases develop. According to Kimura's report, the survival of EBV-T/NK-LPDs from onset was 44%, the median follow-up period was 46 months (9). Unfortunately, chemotherapy that can eradicate EBV-infected T or NK cells for CAEBV has not yet been established. The only effective treatment strategy for a cure currently is allo-HSCT. Fifteen-year OS from onset among the patients treated with allo-HSCT was 60.6%, whereas that of those without allo-HSCT was 25.7% (9). The OS with allo-HSCT was significantly longer than those without allo-HSCT. Furthermore, Kawa et al. retrospectively analyzed the prognosis of patients treated with allo-HSCT in their hospital and reported that the 3-year OS after allo-HSCT among patients receiving reduced intensity conditioning (RIC), was 85%, significantly higher than that of patients receiving myeloablative conditioning (54.5%) (54). These findings indicate that the effects of allo-HSCT were partially due to the replacement and reconstruction of the hematopoietic and immune system by allogeneic grafts, rather than the antitumor effects of chemo- and radiotherapies. In other words, immunological dysfunction plays a pivotal role in the development of CAEBV.

The inflammatory symptoms of CABEV are closely associated with the outcomes. Two reports indicated that patients with active CAEBV had poorer outcomes after allo-HSCT (9, 19). An active disease has been defined as a condition accompanied by any of the following: fever, liver dysfunction, progressive skin lesions, or vasculitis. Condition without any of these clinical findings has been defined as an inactive disease. From reports, it is critical to establish chemotherapy that effectively resolves disease activity. What, then, is the most effective chemotherapy to reduce the activity? Sawada and his colleagues from the Osaka Medical Center and Research Institute for Maternal and Child Health, suggested a sequential treatment strategy consisting of prednisolone, cyclosporine A, and etoposide, a so-called cooling therapy as the first step, followed by combination chemotherapies, CHOP, and ESCAP (55). The last step suggested was RIC followed by allo-HSCT. Unfortunately, the rates of resolution of CAEBV disease activity by the chemotherapies were very low, approximately 10% (our manuscript in preparation). To improve outcomes of CAEBV, it is indispensable to establish a more effective chemotherapy for CAEBV.

As mentioned before, CTL disturbance was indicated for CAEBV (31, 32). Based on these findings, Bollard and her colleagues generated EBV-specific CTLs and used them for the treatment for EBV-positive lymphoid tumors, including T-cell type CAEBV (56, 57). Although significant effects of direct reduction of tumor cells have yet to be achieved, induced CTL infusion has potential as an adjuvant therapy to restore EBV-specific T-cell immunity and prevent disease progression.

Some reagents are candidates for new treatments of CAEBV based on the molecular mechanisms of CAEBV development. As mentioned in the previous section, NF-κB is constitutively activated in EBV-positive T or NK cells and may contribute to suppressed apoptosis of the infected cells (37). A proteasome inhibitor, bortezomib, suppresses NF-κB activation in B-cell neoplasms and was approved as a medicine for multiple myeloma and mantle cell lymphoma. Iwata et al. reported that bortezomib suppressed proliferation and induced apoptosis in EBV-infected cell lines, including T or NK cells (58). The JAK/STAT pathway can also be a target. This pathway is commonly used by multiple cytokine receptors and functions to establish inflammation. As mentioned above, the JAK inhibitor ruxolitinib suppresses the proliferation and cytokine production of EBV-positive T or NK cells. JAK inhibitors are clinically used and effective for cytokine-associated inflammatory diseases, such as RA, UC, and GVHD (59–61). Since CAEBV shows sustained inflammation accompanied by hypercytokinemia, the effects of JAK inhibitors on inflammatory symptoms and disease activity are highly anticipated.

## Summary

CAEBV has been recognized as an endemic disease in East Asia. However, the number of reported cases is increasing worldwide, due to the 2016 WHO classification and the determination of diagnostic criteria. The prognosis of CAEBV is still insufficient. It is necessary to clarify the molecular mechanisms of the disease development to establish effective treatment strategies.

## Author Contributions

AA planned, wrote, and reviewed the manuscript.

### Conflict of Interest Statement

The author declares that the research was conducted in the absence of any commercial or financial relationships that could be construed as a potential conflict of interest.

## References

[1] Epstein MA Achong BG Barr YM. Virus particles in cultured lymphoblasts from burkitt's lymphoma. Lancet (1964) 1:702–3. 10.1016/S0140-6736(64)91524-7 14107961

[2] Swerdlow SH Campo E Pileri SA Harris NL Stein H Siebert R. The 2016 revision of the World Health Organization classification of lymphoid neoplasms. Blood. (2016) 127:2375–90. 10.1182/blood-2016-01-643569 26980727 PMC4874220

[3] ISAACS R. Chronic infectious mononucleosis. Blood (1948) 3:858–61. 18873530

[4] Straus SE. The chronic mononucleosis syndrome. J Infect Dis. (1988) 157:405–12. 10.1093/infdis/157.3.405 2830340

[5] Jones JF Shurin S Abramowsky C Tubbs RR Sciotto CG Wahl R. T-cell lymphomas containing Epstein-Barr viral DNA in patients with chronic Epstein-Barr virus infections. N Engl J Med. (1988) 318:733–41. 10.1056/NEJM198803243181203 2831453

[6] Okano M Kawa K Kimura H Yachie A Wakiguchi H Maeda A. Proposed guidelines for diagnosing chronic active Epstein-Barr virus infection. Am J Hematol. (2005) 80:64–9. 10.1002/ajh.20398 16138335

[7] Ohshima K Kimura H Yoshino T Kim CW Ko YH Lee SS. Proposed categorization of pathological states of EBV-associated T/natural killer-cell lymphoproliferative disorder (LPD) in children and young adults: overlap with chronic active EBV infection and infantile fulminant EBV T-LPD. Pathol Int. (2008) 58:209–17. 10.1111/j.1440-1827.2008.02213.x 18324913

[8] Quintanilla-Martinez L Kimura H Jaffe ES EBV-positive T-cell lymphoproliferative disorders of childhood. In: Jaffe E Harris N Stein H, editors. World Health Organization Classification of Tumors Pathology and Genetics of Tumours of Haematopoietic and Lymphoid Tissues. Lyon: IARC Press (2008), p. 527.

[9] Kimura H Ito Y Kawabe S Gotoh K Takahashi Y Kojima S. EBV-associated T/NK-cell lymphoproliferative diseases in nonimmunocompromised hosts: prospective analysis of 108 cases. Blood (2012) 119:673–86. 10.1182/blood-2011-10-381921 22096243

[10] Cohen JI Jaffe ES Dale JK Pittaluga S Heslop HE Rooney CM. Characterization and treatment of chronic active Epstein-Barr virus disease: a 28-year experience in the United States. Blood (2011) 117:5835–49. 10.1182/blood-2010-11-316745 21454450 PMC3112034

[11] Arai A Imadome KI Watanabe Y Yoshimori M Koyama T Kawaguchi T. Clinical features of adult-onset chronic active Epstein-Barr virus infection: a retrospective analysis. Int J Hematol. (2011) 93:602–9. 10.1007/s12185-011-0831-x 21491104

[12] Kawamoto K Miyoshi H Suzuki T Kozai Y Kato K Miyahara M. A distinct subtype of Epstein-Barr virus-positive T/NK-cell lymphoproliferative disorder: adult patients with chronic active Epstein-Barr virus infection-like features. Haematologica (2018) 103:1018–28. 10.3324/haematol.2017.174177 29242302 PMC6058795

[13] Fujiwara S Kimura H Imadome K Arai A Kodama E Morio T. Current research on chronic active Epstein-Barr virus infection in Japan. Pediatr Int. (2014) 56:159–66. 10.1111/ped.12314 24528553

[14] Asada H Saito-Katsuragi M Niizeki H Yoshioka A Suguri S Isonokami M. Mosquito salivary gland extracts induce EBV-infected NK cell oncogenesis via CD4 T cells in patients with hypersensitivity to mosquito bites. J Invest Dermatol. (2005) 125:956–61. 10.1111/j.0022-202X.2005.23915.x 16297196

[15] Ishihara S Okada S Wakiguchi H Kurashige T Hirai K Kawa-Ha K. Clonal lymphoproliferation following chronic active Epstein-Barr virus infection and hypersensitivity to mosquito bites. Am J Hematol. (1997) 54:276–81. 9092681 10.1002/(sici)1096-8652(199704)54:4<276::aid-ajh3>3.0.co;2-s

[16] Barrionuevo C Anderson VM Zevallos-Giampietri E Zaharia M Misad O Bravo F. Hydroa-like cutaneous T-cell lymphoma: a clinicopathologic and molecular genetic study of 16 pediatric cases from Peru. Appl Immunohistochem Mol Morphol. (2002) 10:7–14. 10.1097/00129039-200203000-00002 11893040

[17] Ito Y Suzuki M Kawada J Kimura H. Diagnostic values for the viral load in peripheral blood mononuclear cells of patients with chronic active Epstein-Barr virus disease. J Infect Chemother. (2016) 22:268–71. 10.1016/j.jiac.2015.11.002 26712582

[18] Kimura H Hoshino Y Kanegane H Tsuge I Okamura T Kawa K. Clinical and virologic characteristics of chronic active Epstein-Barr virus infection. Blood (2001) 98:280–6. 10.1182/blood.V98.2.280 11435294

[19] Arai A Sakashita C Hirose C Imadome KI Yamamoto M Jinta M. Hematopoietic stem cell transplantation for adults with EBV-positive T- or NK-cell lymphoproliferative disorders: efficacy and predictive markers. Bone Marrow Transplant. (2016) 51:879–82. 10.1038/bmt.2016.3 26901705

[20] Suzuki R Yamaguchi M Izutsu K Yamamoto G Takada K Harabuchi Y. Prospective measurement of Epstein-Barr virus-DNA in plasma and peripheral blood mononuclear cells of extranodal NK/T-cell lymphoma, nasal type. Blood (2011) 118:6018–22. 10.1182/blood-2011-05-354142 21984805

[21] Welch JJG Schwartz CL Higman M Chen L Buxton A Kanakry JA. Epstein-barr virus DNA in serum as an early prognostic marker in children and adolescents with Hodgkin lymphoma. Blood Adv. (2017) 1:681–4. 10.1182/bloodadvances.2016002618 29296710 PMC5727814

[22] Chan KCA Woo JKS King A Zee BCY Lam WKJ Chan SL. Analysis of plasma epstein-barr virus DNA to screen for nasopharyngeal cancer. N Engl J Med. (2017) 377:513–22. 10.1056/NEJMoa1701717 28792880

[23] Yoshimori M Imadome K Komatsu H Wang L Saitoh Y Yamaoka S. CD137 Expression is induced by epstein-barr virus infection through LMP1 in T or NK cells and mediates survival promoting signals. PLoS ONE (2014) 9:e112564. 10.1371/journal.pone.0112564 25409517 PMC4237363

[24] Fischer E Delibrias C Kazatchkine MDFischer E, Delibrias C, Kazatchkine MD. Expression of CR2 (the C3dg/EBV receptor, CD21) on normal human peripheral blood T lymphocytes. J Immunol. (1991) 146:865–9. 1703182

[25] Tabiasco J Vercellone A Meggetto F Hudrisier D Brousset P Fournié JJ. Acquisition of viral receptor by NK cells through immunological synapse. J Immunol. (2003) 170:5993–8. 12794126 10.4049/jimmunol.170.12.5993

[26] Stinchcombe JC Bossi G Booth S Griffiths GM. The immunological synapse of CTL contains a secretory domain and membrane bridges. Immunity. (2001) 15:751–61. 11728337 10.1016/s1074-7613(01)00234-5

[27] Imadome K Shimizu N Arai A Miura O Watanabe K Nakamura H. Coexpression of CD40 and CD40 ligand in Epstein-Barr virus-infected T and NK cells and their role in cell survival. J Infect Dis. (2005) 192:1340–8. 10.1086/466530 16170750

[28] Isobe Y Sugimoto K Matsuura I Takada K Oshimi K. Epstein-Barr virus renders the infected natural killer cell line, NKL resistant to doxorubicin-induced apoptosis. Br J Cancer (2008) 99:1816–22. 10.1038/sj.bjc.6604764 18985034 PMC2600699

[29] Anagnostopoulos I Hummel M Kreschel C Stein H. Morphology, immunophenotype, and distribution of latently and/or productively Epstein-Barr virus-infected cells in acute infectious mononucleosis: implications for the interindividual infection route of Epstein-Barr virus. Blood (1995) 85:744–50. 7530505

[30] Arai A Yamaguchi T Komatsu H Imadome K Kurata M Nagata K. Infectious mononucleosis accompanied by clonal proliferation of EBV-infected cells and infection of CD8-positive cells. Int J Hematol. (2014) 99:671–5. 10.1007/s12185-014-1548-4 24643771

[31] Sugaya N Kimura H Hara S Hoshino Y Kojima S Morishima T. Quantitative analysis of Epstein-Barr virus (EBV)-specific CD8+ T cells in patients with chronic active EBV infection. J Infect Dis. (2004) 190:985–8. 10.1086/423285 15295706

[32] Shibayama H Imadome KI Onozawa E Tsuzura A Miura O Koyama T. Virus-specific cytotoxic T cells in chronic active Epstein-Barr virus infection. Rinsho Ketsueki. (2017) 58:583–88. 10.11406/rinketsu.58.583 28679986

[33] Nomura K Kanegane H Otsubo K Wakiguchi H Noda Y Kasahara Y. Autoimmune lymphoproliferative syndrome mimicking chronic active Epstein-Barr virus infection. Int J Hematol. (2011) 93:760–4. 10.1007/s12185-011-0877-9 21626105

[34] Katano H Ali MA Patera AC Catalfamo M Jaffe ES Kimura H. Chronic active Epstein-Barr virus infection associated with mutations in perforin that impair its maturation. Blood (2004) 103:1244–52. 10.1182/blood-2003-06-2171 14576041

[35] Bollard CM Cohen JI. How I treat T-cell chronic active Epstein-Barr virus disease. Blood (2018) 131:2899–05. 10.1182/blood-2018-03-785931 29712633 PMC6024635

[36] Imadome K Shirakata M Shimizu N Nonoyama S Yamanashi Y. CD40 ligand is a critical effector of Epstein-Barr virus in host cell survival and transformation. Proc Natl Acad Sci USA. (2003) 100:7836–40. 10.1073/pnas.1231363100 12805559 PMC164674

[37] Takada H Imadome KI Shibayama H Yoshimori M Wang L Saitoh Y EBV induces persistent NF-κB activation and contributes to survival of EBV-positive neoplastic T- or NK-cells. PLoS ONE (2017) 12:e0174136 10.1371/journal.pone.0174136 28346502 PMC5367708

[38] Nakamura M Iwata S Kimura H Tokura Y. Elevated expression of activation-induced cytidine deaminase in T and NK cells from patients with chronic active Epstein-Barr virus infection. Eur J Dermatol. (2011) 21:780–2. 10.1684/ejd.2011.1433 21697063

[39] Muramatsu M Kinoshita K Fagarasan S Yamada S Shinkai Y Honjo T. Class switch recombination and hypermutation require activation-induced cytidine deaminase (AID), a potential RNA editing enzyme. Cell (2000) 102:553–63. 10.1016/S0092-8674(00)00078-7 11007474

[40] Kotani A Kakazu N Tsuruyama T Okazaki IM Muramatsu M Kinoshita K. Activation-induced cytidine deaminase (AID) promotes B cell lymphomagenesis in Emu-cmyc transgenic mice. Proc Natl Acad Sci USA. (2007) 104:1616–20. 10.1073/pnas.0610732104 17251349 PMC1785248

[41] Kim JH Kim WS Park C. Epstein-Barr virus latent membrane protein 1 increases genomic instability through Egr-1-mediated up-regulation of activation-induced cytidine deaminase in B-cell lymphoma. Leuk Lymphoma (2013) 54:2035–40. 10.3109/10428194.2013.769218 23363221

[42] Okuno Y Murata T Sato Y Muramatsu H Murakami N Okuno T Genetic background of chronic active Epstein-Barr virus disease. Blood (2017) 130:1468.28760889

[43] Jiang L Gu ZH Yan ZX Zhao X Xie YY Zhang ZG. Exome sequencing identifies somatic mutations of DDX3X in natural killer/T-cell lymphoma. Nat Genet. (2015) 47:1061–6. 10.1038/ng.3358 26192917

[44] Schmitz R Young RM Ceribelli M Jhavar S Xiao W Zhang M. Burkitt lymphoma pathogenesis and therapeutic targets from structural and functional genomics. Nature (2012) 490:116–20. 10.1038/nature11378 22885699 PMC3609867

[45] Cai W Xiong Chen Z Rane G Satendra Singh S Choo Z Wang C. Wanted DEAD/H or alive: helicases winding up in cancers. J Natl Cancer Inst. (2017) 109. 10.1093/jnci/djw278 28122908

[46] Arai A Nogami A Imadome K Kurata M Murakami N Fujiwara S. Sequential monitoring of serum IL-6, TNF-α, and IFN-γ levels in a CAEBV patient treated by plasma exchange and immunochemotherapy. Int J Hematol. (2012) 96:669–73. 10.1007/s12185-012-1170-2 22983646

[47] Onozawa E Shibayama H Imadome KI Tsuzura A Koyama T Miura O. Inflammatory cytokine production in chronic active Epstein-Barr virus infection. Rinsho Ketsueki (2017) 58:189–196. 10.11406/rinketsu.58.189 28381684

[48] Hanada T Yoshimura A. Regulation of cytokine signaling and inflammation. Cytokine Growth Factor Rev. (2002) 13:413–21. 10.1016/S1359-6101(02)00026-6 12220554

[49] Scott LM Gandhi MK. Deregulated JAK/STAT signalling in lymphomagenesis, and its implications for the development of new targeted therapies. Blood Rev. (2015) 29:405–15. 10.1016/j.blre.2015.06.002 26123794

[50] Arora L Kumar AP Arfuso F Chng WJ Sethi G. The role of signal transducer and activator of transcription 3 (STAT3) and its targeted inhibition in hematological malignancies. Cancers (Basel) (2018) 10:E327. 10.3390/cancers10090327 30217007 PMC6162647

[51] Onozawa E Shibayama H Takada H Imadome KI Aoki S Yoshimori M. STAT3 is constitutively activated in chronic active Epstein-Barr virus infection and can be a therapeutic target. Oncotarget (2018) 9:31077–89. 10.18632/oncotarget.25780 30123428 PMC6089567

[52] Chen H Hutt-Fletcher L Cao L Hayward SD. A positive autoregulatory loop of LMP1 expression and STAT activation in epithelial cells latently infected with Epstein-Barr virus. J Virol. (2003) 77:4139–48. 10.1128/JVI.77.7.4139-4148.2003 12634372 PMC150666

[53] Nosbaum A Prevel N Truong HA Mehta P Ettinger M Scharschmidt TC. Cutting edge: regulatory T cells facilitate cutaneous wound healing. J Immunol. (2016) 196:2010–4. 10.4049/jimmunol.1502139 26826250 PMC4761457

[54] Kawa K Sawada A Sato M Okamura T Sakata N Kondo O. Excellent outcome of allogeneic hematopoietic SCT with reduced-intensity conditioning for the treatment of chronic active EBV infection. Bone Marrow Transplant. (2011) 46:77–83. 10.1038/bmt.2010.122 20498651

[55] Sawada A Inoue M Kawa K. How we treat chronic active Epstein-Barr virus infection. Int J Hematol. (2017) 105:406–18. 10.1007/s12185-017-2192-6 28210942

[56] Bollard CM Gottschalk S Leen AM Weiss H Straathof KC Carrum G. Complete responses of relapsed lymphoma following genetic modification of tumor-antigen presenting cells and T-lymphocyte transfer. Blood (2007) 110:2838–45. 10.1182/blood-2007-05-091280 17609424 PMC2018666

[57] Yonese I Takase H Yoshimori M Onozawa E Tsuzura A Miki T. CD79B mutations in primary vitreoretinal lymphoma: diagnostic and prognostic potential. Eur J Haematol. (2018) 102:191–6. 10.1111/ejh.13191 30390359

[58] Iwata S Yano S Ito Y. Bortezomib induces apoptosis in T lymphoma cells and natural killer lymphoma cells independent of Epstein-Barr virus infection. Int J Cancer (2011) 129:2263–73. 10.1002/ijc.25873 21170988

[59] Malemud CJ. The role of the JAK/STAT signal pathway in rheumatoid arthritis. Ther Adv Musculoskelet Dis. (2018) 10:117–27. 10.1177/1759720X18776224 29942363 PMC6009092

[60] Kasembeli MM Bharadwaj U Robinson P Tweardy DJ. Contribution of STAT3 to inflammatory and fibrotic diseases and prospects for its targeting for treatment. Int J Mol Sci. (2018) 19:E2299. 10.3390/ijms19082299 30081609 PMC6121470

[61] Gupta V Hari P Hoffman R. Allogeneic hematopoietic cell transplantation for myelofibrosis in the era of JAK inhibitors. Blood (2012) 120:1367–79. 10.1182/blood-2012-05-399048 22700718 PMC5800543

